# Book Reviews

**Published:** 1876-03

**Authors:** 


### Book Reviews.

[NOTE.—All works reviewed in the pages of the CHICAGO MEDICAL JOURNAL AND EXAMINER may be found in the extensive stock of W. B. KEEN, COOKE & Co., whose catalogue of Medical Books will be sent to any address upon request.]

A SERIES OF AMERICAN CLINICAL LECTURES. Edited by *E. C. Seguin, M.D.* New York: G. P. Putnam's Sons.

We have before us the last two numbers of this most excellent series of lectures, that have been running through the year 1875, appearing in pamphlet, and paged and arranged for binding in book form.

The lectures of the series are as follows: *On Disease of the Hip Joint*, SAYRE; *Acute Rheumatism in Infancy and Childhood*, JACOBI; *Pneumothorax*, FLINT; *Rest in the Treatment of Nervous Diseases*, MITCHELL; *On the Treatment of Sciatica*, THOMSON; *Otitis*, AGNEW; *Capillary Bronchitis of Adults*, ELLIS; *Inflammatory Origin of Phthisis*, HUTCHINSON; *Peritonitis*, LOOMIS; *Gleet, and its Relations to Urethral Stricture*, OTIS; *On the Diagnosis of Paraplegia*, WOOD, Jr.; and *On the Nature of the Gouty Vice*, DRAPER.

Most of these lectures are excellent; many of them are superb in their terseness and the clinical value of the points set forth. Because we are in need of more purely clinical studies by men best able to make them, we regard the present effort worthy the endorsement and fostering of the profession.

The eleventh lecture on the diagnosis of paraplegia by Dr. H. C. Wood, Jr., is one of the most clear and plain expositions of the subject we remember to have seen. It is poetical in the truths it tells in few words. The whole volume might well be purchased for this one lecture.

The closing lecture On the Nature of the Gouty Vice, by Dr. W. H. Draper, is peculiar. This seems to be a production, the chief object of which is to establish the existence in large masses of the community of a gouty vice.

We had supposed gout an unusual disorder in this country. According to the teaching of this lecture most people who are sick are victims of gouty vice.

If your ancestors had rheumatism, anything that ails you is likely to be the vice of gout; if you chance to have aches in your joints or tendons, it is due to gout. If you have a sour stomach, feelings of malaise, irregular action of the bowels; if you have what the world calls biliousness, if you have flatulent and acid dyspepsia, it is due to the disturbances of the gouty vice. If you have with these digestive disturbances any eruption on the skin, it is sure to be due to the gout that is in you; it is doubly sure to be so if the disorder chance to be psoriasis, chronic eczema or acne. We are told that frontal headache, pain in the back of the head and neck, pain in the eyeballs, burning sensations in the palms and soles, numbness of the hands and forearms, pains in the tendo achillis or dorsum of the foot, "are some of the more common dysæsthesiæ" of gouty subjects, and that cynanche tonsillaris, acute pneumonia and cirrhosis of kidney and liver, tend to occur oftener in gouty than other subjects.

In the diagnosis the argument is not toward learning what ails the patient, but toward proving the existence of the gouty vice. "To substantiate the gouty nature of a cutaneous lesion, you must look for some of the concomitant functional and lesional derangements" of gout. "Frequently valuable information, corroborating a suspicion of this vice, may be obtained by seeking the cause of death in the family," etc. "Death from 'heart disease,' 'Bright's disease,' 'dropsy,' or sudden death from apoplexy, are always significant circumstances in the history of the gouty diathesis, just as death from phthisis is presumptive evidence of a scrofulous taint."

Reading this lecture we gather that most of us eat too much or stimulate too much with fermented liquor, or we do not get enough oxygen in the air we breathe. There results a failure of perfect oxidation of the waste of food and tissue, and what should become urea stops short at uric acid. This in the blood leads to all the lesions mentioned, and many more. Whenever any of these ills come upon us, it is gout, and we have the gouty vice.

But why call it gout, and make the word a scapegoat to carry such burdens? Any other word would do as well, and many would answer better. Why not say rheumatic, bilious or acid diathesis? or the dyspeptic diathesis, which would be far better, more philosophical, and not half so misleading?

The treatment recommended is: oxygen, iron and dilute alkalies.

THE PHYSICIANS' COMBINED CALL-BOOK AND TABLET. Prepared by *Dr. Ralph Walsh*, of Washington, D. C.

This is a book designed to take the place of the ordinary visiting list of physicians. It is long, thin and flat, and so easier carried in the pocket than most of our lists. It may be used continuously until full, not being prepared for any particular year and month.

A MANUAL OF BANDAGING, ADAPTED FOR SELF-INSTRUCTION. By *C. Henri Leonard, A.M., M.D.* With over One Hundred Illustrations. Detroit. 1876.

The author has shown admirable endurance in the tedious and dreary work of compiling a manual on bandaging. It is designed for the self-instruction of those who have never had the advantage of hospital drill; but we fear the compilation is too indiscriminate for the purpose. It describes the bandages of all patterns, simple and complicated, modern and obsolete, practical and ornamental, useful and useless.

One might as well attempt to learn dancing from books, as to acquire the art of bandaging by perusing a manual.

